# The Glutamine Synthetases Are Required for Sensory Hair Cell Formation and Auditory Function in Zebrafish

**DOI:** 10.3390/ijms252111561

**Published:** 2024-10-28

**Authors:** Yuanrong Zhao, Ziyang Wang, Mengting Xu, Fuping Qian, Guanyun Wei, Dong Liu

**Affiliations:** Nantong Laboratory of Development and Diseases, School of Life Sciences, Co-Innovation Center of Neuroregeneration, Nantong University, Qixiu Road 19, Nantong 226001, China; z1024119424@163.com (Y.Z.); biowzy@163.com (Z.W.); amyxu.mt@foxmail.com (M.X.); qianfuping198911@163.com (F.Q.)

**Keywords:** hair cells, glutamine synthetases, apoptosis, hearing, zebrafish

## Abstract

The development of sensory hair cells (HCs) is closely linked to hearing loss. There are still many unidentified genes that may play a crucial role in HC development and function. Glutamine synthetase, Glul, is expressed in sensory hair cells and auditory organs. However, the role of the *Glul* gene family in the auditory system remains largely unexplored. This study aims to investigate the function of the *Glul* gene family in the auditory system. The expression patterns of the *glul* gene family were examined via in situ hybridization in zebrafish embryos. It was revealed that the expression of *glula* occurred in the otic vesicle, while *glulb* was expressed in the neuromast. In contrast, *glulc* did not exhibit any discernible signal. *glula* loss of function caused abnormal otolith formation and reduced hair cell number in otic vesicles, while *glulb* knockdown caused a decrease in HC number in both neuromasts and otic vesicles and impaired auditory function. Furthermore, we found that the knockdown of *glulb* induces apoptosis of hair cells. Transcriptomic analysis of zebrafish with *glula* and *glulb* knockdown revealed significant alterations in the expression of many genes associated with auditory organs. The current study sheds light on the requirement of *glula* and *glulb* in zebrafish hair cell formation and auditory function.

## 1. Introduction

Hearing loss represents a prevalent sensory disorder in humans, significantly impacting social interactions and communication and causing substantial physical and psychological distress [1,2]. Impairment or loss of hair cells during development can lead to hearing loss. HCs are specialized sensory cells that capture external stimuli and convert them into neural signals, enabling animals to perceive auditory, balance, and hydrodynamic information. In vertebrates, HCs are located in the auditory and vestibular organs of the inner ear, while in fish and aquatic amphibians, they are also found in the lateral line organs [3]. 

Zebrafish are frequently employed to investigate the mechanisms underlying the development of HCs. Zebrafish embryos and larvae offer a unique advantage due to their transparency, enabling real-time observation and in vivo dynamic imaging of HC development. Additionally, their rapid developmental timeline accelerates research cycles. In adult zebrafish, the inner ear comprises three semicircular canals—the anterior, posterior, and horizontal canals—along with otolith organs, including the elliptic sac, balloon, and auditory ampulla. These structures contribute to vestibular balance and auditory perception [4].

Moreover, the lateral line system consists of multiple neuromasts and lateral line HCs on the body surface, which share similarities with inner ear HCs [5]. The differentiation, development, and functional maturation of HCs are intricate processes influenced by various signaling pathways, transcription factors, secretory factors, and growth factors. Utilizing zebrafish as a research tool to investigate HCs is a widely adopted approach in scientific studies.

*Glutamine synthetase (Glul)* is a crucial enzyme in nitrogen metabolism and regulating ammonia levels. Its pivotal function involves catalyzing the ATP-dependent conversion of glutamate and ammonia into glutamine, a key precursor for protein synthesis. *Glul* is essential in mitigating the toxicity of excessive free ammonium ions, which accumulate during certain biological processes and prove harmful to body health. It has been discovered that *Glul* is involved in diverse processes, including the regulation of ammonia metabolism in the brain and liver, cell proliferation, cell migration, and cancer development [6,7,8]. *Glul* has been implicated in the increased risk of cardiovascular diseases among individuals with type 2 diabetes and is closely associated with the familial inheritance of hepatitis B and hepatocellular carcinoma [9,10]. Furthermore, the knockout of *Glul* resulted in significant reductions in proliferation capacity and the inhibition of *p38 MAPK* and *ERK1/ERK2* signaling pathways in SK-BR-3 cells [8]. Downregulation of *Glul* has also been shown to impede the migration of human umbilical vein endothelial cells [7]. In zebrafish, it was revealed that the expression levels of *glul* (*glula*, *glulb*, and *glulc*) mRNA were upregulated in various tissues, including the brain, liver, and kidney, in response to hyperammonemia stress [11]. However, despite these extensive investigations, the role of *Glul* in the auditory system remains largely unexplored. Glutamine synthetase, Glul, is expressed in sensory hair cells and auditory organs. Therefore, we hypothesize that the *Glul* gene family might play important roles in the auditory system. This study aims to investigate the function of the *Glul* gene family in auditory formation and function.

The present study highlights the role of *glula* and *glulb* in the regulation of hair cell formation and auditory function.

## 2. Results

### 2.1. The Glula and Glulb Genes Are Expressed in the Otic Vesicles and Neuromasts of Zebrafish

**Figure 1:**
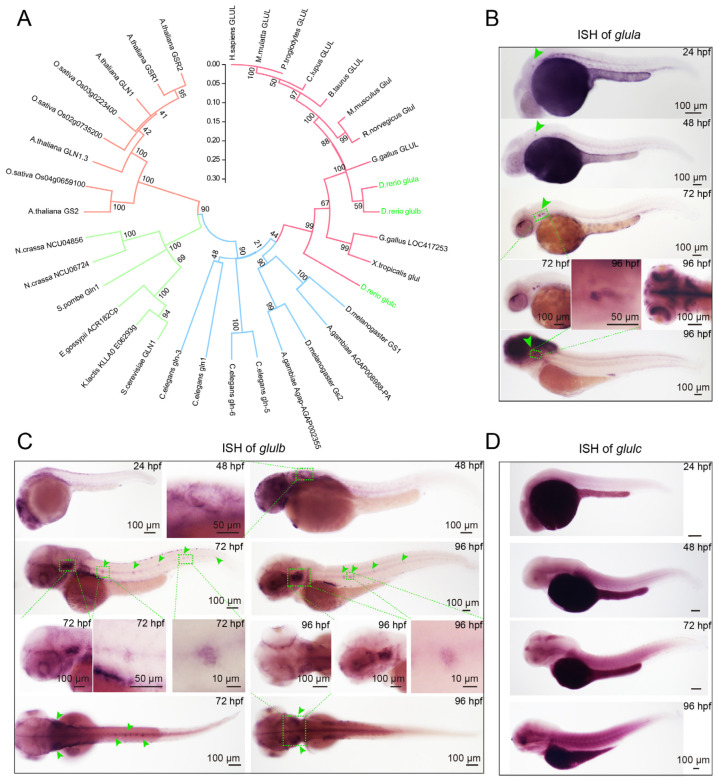
Phylogenetic analysis of *Glul* across diverse species and the expression patterns of the *Glul* gene family in zebrafish. (**A**) The MEGA software (Version 11.0.13) was utilized to construct a phylogenetic tree based on amino acid sequences, encompassing a diverse range of species including *H. sapiens*, *P. troglodytes*, *M. mulatta*, *C. lupus*, *B. taurus*, *M. musculus*, *R. norvegicus*, *G. gallus*, *D. rerio*, *D. melanogaster*, *A. gambiae*, *C. elegans*, *S. cerevisiae*, *K. lactis*, *E. gossypii*, *S. pombe*, *N. crassa*, *A. thaliana*, *O. sativa*, and *X. tropicalis.* Branches of the same color are closer. Whole-mount in situ hybridization (WISH) was performed using *glula*, *glulb*, and *glulc* probes in wild-type zebrafish. (**B**) The results demonstrated that the expression of the *glula* gene was observed specifically in the otic vesicle. Green arrowheads indicate otic vesicle. Boxes indicate the magnified regions. (**C**) while the *glulb* gene exhibited expression in both the otic vesicle and lateral line. Green arrowheads indicate otic vesicle and neuromasts. Boxes indicate the magnified regions. (**D**) Conversely, no significant signal was detected for the *glulc* gene.

To investigate the conservation of the *Glul* gene, we constructed an evolutionary tree comprising 22 species based on their amino acid sequences. The findings demonstrate widespread conservation of the *Glul* gene across multiple species (Figure 1A). In zebrafish, the *glul* gene family contains three paralogs: *glula*, *glulb*, and *glulc*. Multiple sequence alignment analyses of Glula (NP_853537.1), Glulb (NP_878286.3), and Glulc (NP_001068582.1) revealed 89.2% (Glula vs. Glulb), 70.2% (Glula vs. Glulc), and 70.8% (Glulb vs. Glulc) identity, respectively. To investigate the spatiotemporal expression patterns of the *glul* gene family during zebrafish embryonic development, we conducted whole embryo in situ hybridization (WISH) using specific antisense probes targeting *glula, glulb,* and *glulc*, respectively. The results demonstrated the expression of *glula* in various tissues, including the inner ear, notochord, nervous system, and caudal vein plexus in zebrafish. The *glula* expression was detectable as early as 24 hpf in the inner ear (Figure 1B). The expression of *glulb* was observed in the inner ear, brain, and lateral line neuromasts of zebrafish (Figure 1C). Importantly, *glulb* expression was detectable in the zebrafish brain as early as 24 hpf. By 48 hpf, *glulb* expression emerged in the otic vesicle. Subsequently, *glulb* exhibited expression in the inner ear and lateral line neuromasts by 72 hpf. However, no significant signal of *glulc* expression was observed (Figure 1D). These findings suggest that both *glula* and *glulb* are involved in zebrafish inner ear and sensory hair cell development.

### 2.2. Knockdown of Glula Reduced Survival Rates and Abnormal Otolith Development in Zebrafish

**Figure 2:**
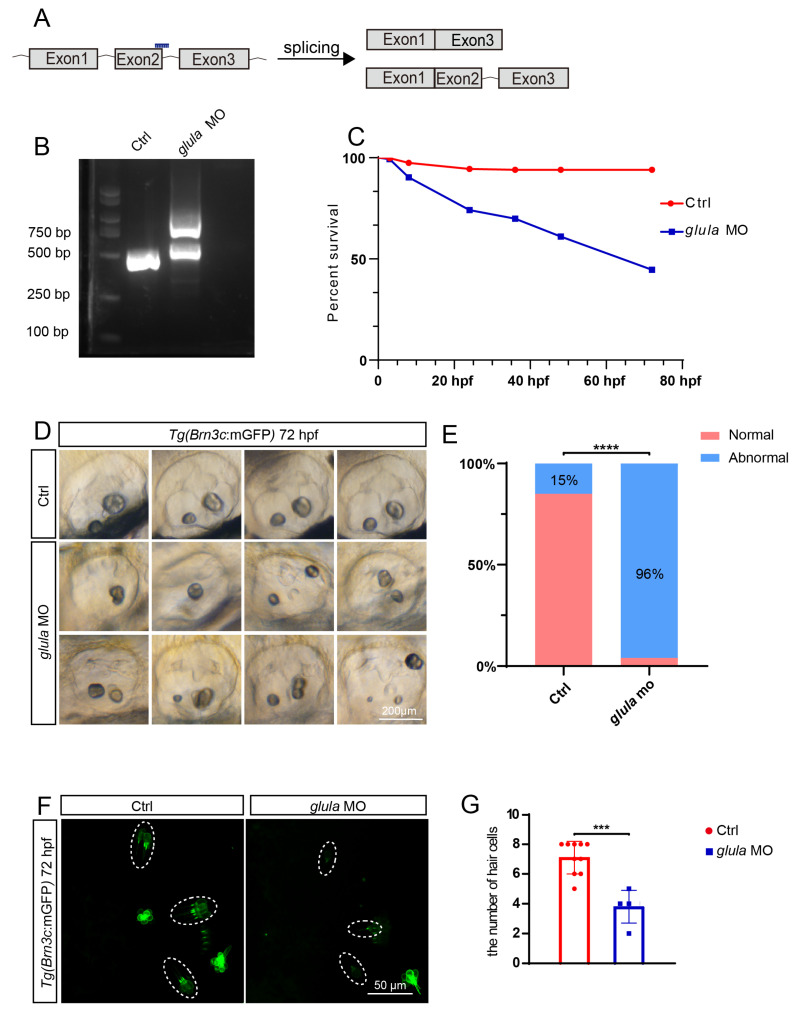
The downregulation of *glula* results in a reduced survival rate and impacts otolith development. (**A**) Diagram illustrating the *glula*-MO binding site and potential cleavage methods. (**B**) Reproducible knockdown of the *glula* gene was successfully demonstrated through PCR experimentation. (**C**) Knockdown of *glula* leads to a significant decrease in the survival rate of zebrafish. (**D**) Abnormal otolith development was observed in zebrafish following *glula*-MO injection. (**E**) Statistical analysis revealed that 98% of zebrafish injected with *glula*-MO exhibited aberrant otolith development. Chi-squared test: ****, *p* < 0.0001. (**F**) After the injection of *glula*-MO, a significant decrease in hair cells was observed in otic vesicles. Dash line cycles indicate the crista hair cells. (**G**) Statistical analysis of the hair cell number in otic vesicles. *t*-test: ***, *p* < 0.001.

To investigate the role of *glula* in zebrafish embryonic development, we designed a specific morpholino targeting *glula* (*glula*-MO). Subsequently, we generated a zebrafish model with downregulated *glula* expression by microinjecting the *glula*-MO into the embryos. The total RNA from 72-hour-old *glula*-MO zebrafish embryos was extracted for PCR analysis, which revealed aberrant splicing events in these embryos (Figure 2A,B). Further examination of embryonic development demonstrated that, compared to the control group, zebrafish embryos injected with *glula*-MO exhibited lower survival rates and a higher incidence of morphological abnormalities among the surviving individuals (Figure 2C). Notably, abnormal otolith development was observed in zebrafish embryos injected with *glula*-MO, including variations in otolith number (decrease or increase), fusion of otoliths, and irregular distribution patterns (Figure 2D). Analysis of 100 zebrafish otic vesicles showed that while only 15% of control zebrafish displayed abnormal otolith development, this percentage increased significantly to 96% in *glula*-morphants (Figure 2E). Our findings indicate that knockdown of *glula* expression leads to reduced survival rates and impaired otolith development in zebrafish embryos. The transgenic zebrafish line *Tg(Brn3c: mGFP)* specifically labels the zebrafish hair cells and certain parts of the retinal ganglion neurons. Embryos from these two transgenic lines were collected and injected with *glula*-MO at the 1–2 cell stage. Subsequently, confocal microscopy imaging analysis was employed to examine the phenotypes of sensory hair cells in otic vesicles, including three cristae of the inner ear (PC: posterior cristae; LC: lateral cristae; AC: anterior cristae). We found a decrease in hair cell numbers in *glula*-MO-injected zebrafish embryos compared to the control group counterparts within the three cristae (Figure 2F,G).

### 2.3. Repression of Glulb Expression Elicits Aberrant Behavioral Patterns in Zebrafish

**Figure 3:**
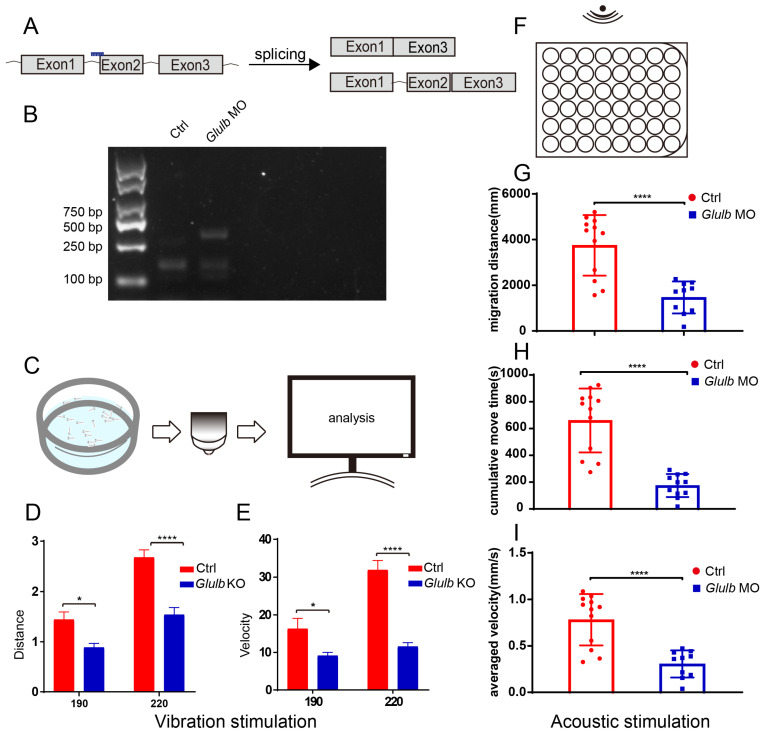
The knockdown of *glulb* expression resulted in a delayed response of zebrafish to both vibrational and auditory stimuli. (**A**) Schematic representation of the *glulb*-MO binding site and potential cleavage methodologies. (**B**) Successful *glulb* gene knockdown validated by PCR analysis. (**C**) Diagram illustrating the experimental setup for vibration stimulation: 7-day-old juvenile zebrafish were positioned within the mold, exposed to vibration stimulation, and subsequently subjected to data recording and analysis. (**D**,**E**) Statistical analysis revealed a significant decrease in the distance traveled and swimming velocity of zebrafish in the *glulb*-MO group compared to the Ctrl group. *t*-test: *, *p* < 0.05; ****, *p* < 0.0001. (**F**) Five-day-old zebrafish juveniles were placed in the orifice plate, exposed to acoustic stimulus, and subjected to subsequent data recording and analysis. (**G**–**I**) The *glulb*-MO group exhibited a significant reduction in the movement distance, average speed, and sustained movement time of zebrafish compared to the control group, as revealed by statistical analysis. *t*-test: ****, *p* < 0.0001.

To investigate the role of *glulb* in zebrafish embryonic development, we generated a zebrafish model with the *glulb* downregulated by introducing a specific morpholino targeting *glulb* (*glulb*-MO) into zebrafish embryos. The *glulb*-MO was injected into *Tg(Brn3c:mGFP)* zebrafish embryos at the 1–2 cell stage, and the phenotypes of these embryos were observed using microscopy at 72 hpf. Embryos exhibiting consistent phenotypes were selected for further analysis. Total RNA was extracted from these embryos, and PCR confirmed erroneous splicing in zebrafish embryos injected with the *glulb*-MO (Figure 3A,B).

To investigate potential alterations in response to the vibration stimulation following *glulb* knockdown, we conducted a vibration stimulation experiment. A total of 20 randomly selected zebrafish, at the stage of 7 days post-fertilization (dpf), were placed in a mold and subjected to vibration stimulation while their responses were recorded using high-definition cameras (Figure 3C). The swimming distance and speed of the zebrafish in the control group (Ctrl) and *glulb*-MO group were measured under stimuli intensities of 190 and 220, respectively. Notably, a significant decrease was observed in the *glulb*-MO group at an intensity of 220, whereas no significant difference was noted at an intensity of 190 (Figure 3D,E). Furthermore, to assess the response to sound stimulation among zebrafish at the stage of 5 dpf in both the Ctrl and *glulb*-MO groups, they were placed within an orifice with sound stimulation administered every five minutes (Figure 3F). Statistical analysis demonstrated a substantial reduction in moving distance, average speeds, and continuous movement time among zebrafish within the *glulb*-MO group compared to the Ctrl group (Figure 3G–I). Taken together, the knockdown of *glulb* resulted in a diminished sensitivity of zebrafish towards both vibration and sound stimuli.

Additionally, we generated a zebrafish model with *glulb* knockout (*glulb*-KO) using CRISPR/Cas9 technology by targeting the translation start site near the first exon (Supplementary Figure S1A). Sequencing analysis of zebrafish co-injected with Cas9 mRNA and sgRNA revealed multiple peaks downstream of the sgRNA site, indicating successful targeting. The *glulb*-KO mutation was confirmed (Supplementary Figure S1B). Subsequent vibration stimulation experiments showed no significant difference in swimming distance and speed between the control group and *glulb*-KO group at an intensity of 190. However, at an intensity of 220, the *glulb*-KO group had a significantly reduced swimming distance and speed (Supplementary Figure S1C,D).

### 2.4. Knockdown of Glulb Leads to Aberrant Hair Cell Development in Zebrafish

**Figure 4:**
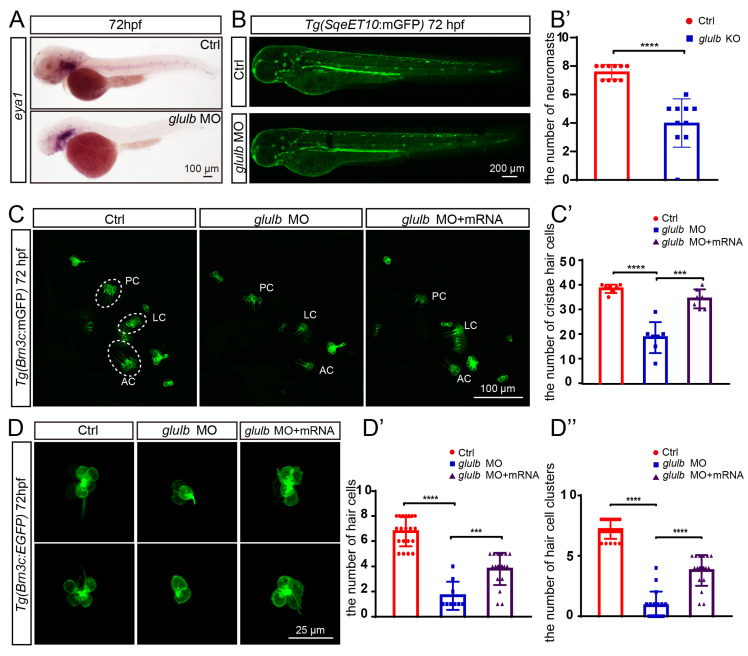
The zebrafish that were injected with *glulb*-MO exhibited a significant decrease in the number of neuromasts, hair cell clusters, and hair cells, and co-injection of *glulb* mRNA successfully rescued the observed phenotype following *glulb* knockdown. (**A**) In situ hybridization using the *eya1* probe revealed a reduced number of neuromasts in the *glulb*-MO zebrafish group compared to the Ctrl group at 72 hpf. (**B**) The application of laser confocal microscopy revealed a decrease in the number of neuromasts in *glulb*-MO zebrafish embryos at 72 hpf compared to the control zebrafish, and (**B’**) the observed discrepancy exhibited statistical significance. *t*-test: ****, *p* < 0.0001. (**C**) The number of hair cells in the inner ear of *glulb*-MO zebrafish exhibited a significant decrease compared to the control group. Co-injection of *glulb* mRNA effectively rescued this phenotype, and (**C’**) subsequent statistical analysis revealed statistically significant differences. One-way ANOVA: ****, *p* < 0.0001; ***, *p* < 0.001. (**D**) The *glulb*-MO zebrafish exhibited decreased hair cells within the L3 neuromasts, which can be rescued by co-injection of *glulb* mRNA. (**D’**) Statistical analysis revealed a significant difference. (**D”**) A statistical analysis of hair cell clusters in the control, *glulb*-MO injected, and *glulb*-MO co-injection with *glulb* mRNA embryos was conducted. One-way ANOVA: ****, *p* < 0.0001; ***, *p* < 0.001.

The knockdown of *glulb* expression resulted in a decrease in the number of neuromasts and hair cell clusters and inner ear development in zebrafish (Figure 4). The *eya1* probe was utilized for specific labeling of zebrafish neuromasts, which were injected into *glulb*-MO zebrafish at the one-cell stage. Subsequently, a whole-mount in situ hybridization (WISH) experiment was conducted, revealing a significant reduction in the number of neuromasts in *glulb*-MO zebrafish (Figure 4A). Moreover, *glulb*-MO was injected into *Tg(SqeET10:mGFP)* zebrafish embryos at the one-cell stage, which specifically labels the zebrafish lateral line, and confocal microscopic analysis revealed a significant reduction in the number of neuromasts in the lateral line when the zebrafish developed to 72 hpf (Figure 4B), further confirmed by statistical analysis (Figure 4B’). The *Tg(Brn3c:mGFP)* zebrafish, specifically labeling zebrafish hair cells, was employed to investigate the impact of *glulb* on zebrafish hair cells. The number of hair cells in the three cristae was decreased in zebrafish embryos following *glulb*-MO injection, and co-injecting *glulb* mRNA partially rescued the phenotype (Figure 4C,C’). Simultaneously, the hair cell numbers of L3 neuromasts in the zebrafish lateral line were concurrently observed and subjected to statistical analysis. The results revealed a significant reduction in the number of hair cells, while co-injecting *glulb* mRNA partially rescued this phenotype (Figure 4D,D’), as well as restoring the number of hair cell clusters in the zebrafish lateral line (Figure 4D”).

The *glulb* knockout model exhibited consistent results with the knockdown findings (Supplementary Figure S2). Co-injection of Cas9 mRNA and *glulb* sgRNA was performed at the one-cell stage in *Tg(Brn3c:mGFP)* zebrafish. The zebrafish were observed at 72 h of development, revealing a significant decrease in the number of hair cell clusters (Supplementary Figure S2A,A’). Additionally, a significant reduction in the number of hair cells was observed in the three cristae and L3 neuromasts in the *glulb*-KO model, indicating a pronounced impact on sensory organ development (Supplementary Figure S2B,C). Moreover, there was also a statistically significant decrease in the number of hair cells observed in the three cristae and L3 neuromasts of the *glulb*-KO model (Supplementary Figure S2B’,C’). In line with the findings following *glulb* knockdown, this study provides further validation that downregulation of *glulb* expression results in aberrant hair cell development in zebrafish.

### 2.5. Knockdown of Glulb Resulted in Apoptosis in Zebrafish Hair Cells, and Supplementation with Glutamine Failed to Rescue This Effect

**Figure 5:**
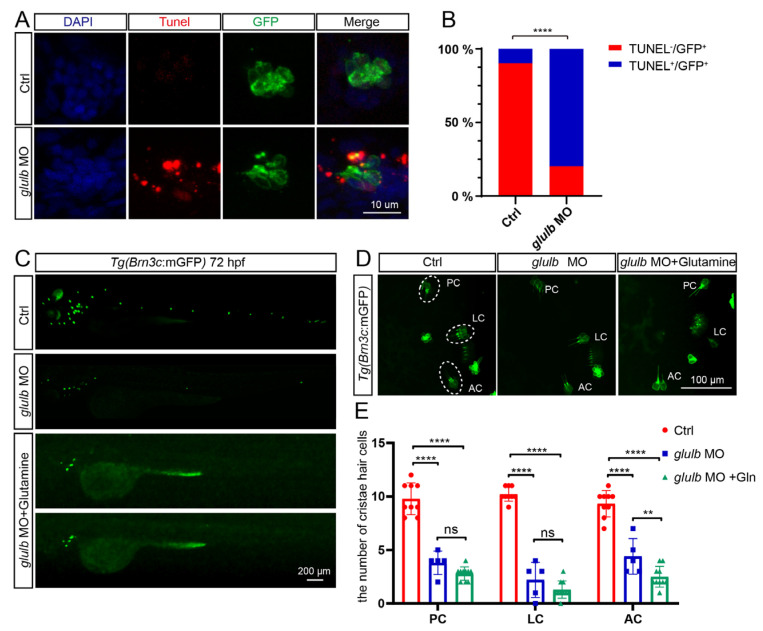
Knockdown of *glulb* induces apoptosis in zebrafish hair cells, and the supplementation of glutamine fails to rescue the observed phenotype. (**A**) The zebrafish embryos treated with *glulb*-MO and the control zebrafish were subjected to TUNEL staining, which revealed the presence of apoptotic signals, specifically in the hair cells of the *glulb*-MO-treated zebrafish. (**B**) The percentage of hair cells displaying apoptotic signals was evaluated in control zebrafish embryos and *glulb*-MO-injected zebrafish. TUNEL+/GFP+ denotes hair cells exhibiting apoptotic signals, while TUNEL-/GFP+ represents hair cells lacking apoptotic signals. Chi-squared test: ****, *p* < 0.0001. (**C**) The phenotype of hair cell clusters in whole embryonic zebrafish was visualized using confocal laser microscopy. Significantly reduced hair cell clusters were observed in *glulb*-MO-treated zebrafish compared to Ctrl zebrafish, and co-injection of *glulb*-MO and glutamine failed to rescue the reduction in hair cell clusters. (**D**) Similarly, a decreased number of hair cells was observed in the inner ear of zebrafish embryos treated with *glulb*-MO compared to control zebrafish, and co-injection of *glulb*-MO and glutamine failed to rescue the phenotype. Lateral crista (LC), anterior crista (AC) and posterior crista (PC). (**E**) The number of hair cells exhibited a significant reduction statistically, with no substantial rescue observed following glutamine supplementation. One-way ANOVA: ****, *p* < 0.0001; **, *p* < 0.01, ns means no significant.

The TUNEL (terminal deoxynucleotidyl transferase-mediated nick end labeling) staining technique was employed to label the 3′-OH ends of fragmented DNA in apoptotic cells. Notably, apoptotic signals were observed specifically in the hair cells of *glulb* knockdown zebrafish (Figure 5A). Subsequent statistical analysis revealed that more than eighty percent of GFP+ cells exhibited positive TUNEL signals (Figure 5B), indicating that most hair cells underwent apoptosis.

Given the observed decrease in glutamine synthesis following *glulb* knockdown, we investigated the potential impact of glutamine on hair cell development. To address this, a co-injection approach was employed to supplement glutamine for *glulb*-MO zebrafish at the one-cell stage. The results revealed that adding glutamine did not rescue the phenotype characterized by reduced hair cell clusters in the lateral line (Figure 5C) or the loss of hair cells in the inner ear (Figure 5D). Statistical analysis demonstrated that supplementation with glutamine had no rescuing effect (Figure 5E). These findings indicate that reduced hair cells in zebrafish following *glulb* knockdown cannot be attributed to a decrease in glutamine levels.

### 2.6. Transcriptomic Analysis of Glula or Glulb Knockdown in Zebrafish

**Figure 6:**
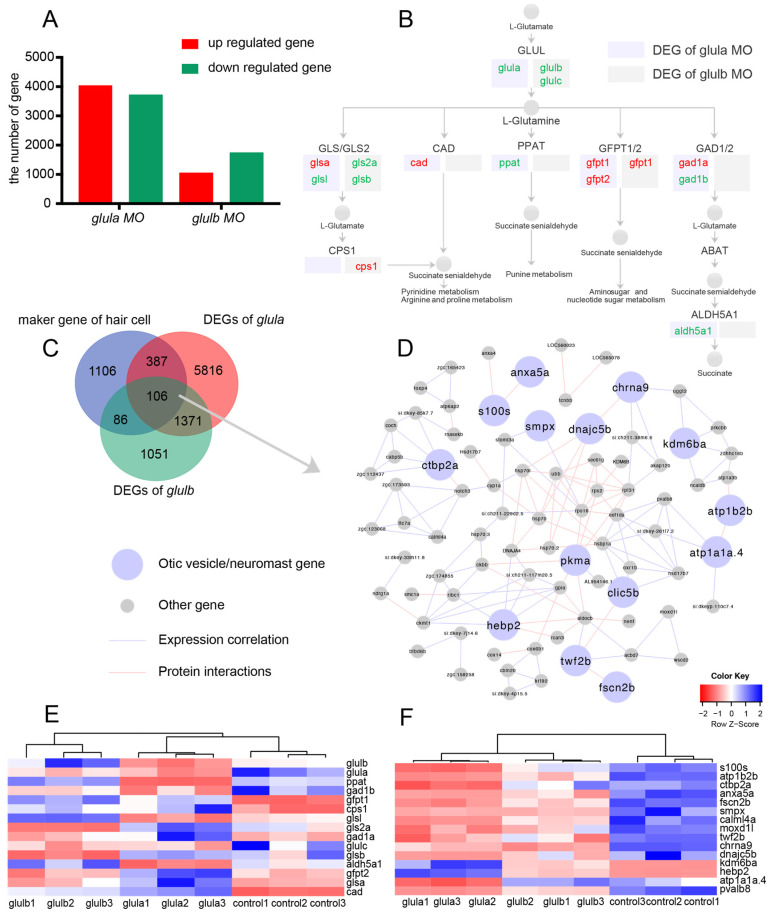
Transcriptomic analysis of zebrafish with *glula* knockdown and *glulb* knockdown. (**A**) Differentially expressed genes (DEGs) in the transcriptome of *glula* knockdown zebrafish and *glulb* knockdown zebrafish were identified. (**B**) DEGs in the glutamine pathway were observed in the transcriptomes of *glula* knockdown and *glulb* knockdown zebrafish. (**C**) The overlap of DEGs in *glula* transcriptomes, *glulb* transcriptomes, and the hair cell marker genes was identified through single-cell sequencing. (**D**) In the gene interaction network of overlap DEGs, pink nodes represent gene expression in the neuromasts, purple nodes indicate gene expression in the otic vesicles, gray nodes denote other genes, purple edges signify gene expression correlation, and pink edges indicate protein interactions. (**E**) Heatmaps illustrating DEGs in the *glul* pathway. (**F**) Heatmaps of DEGs in neuromasts and otic vesicles within the gene–gene interaction network.

To investigate global changes in gene expression following the knockdown of *glula* or *glulb* in zebrafish, we employed RNA-sequencing to analyze the whole-genome transcriptomes of *glula* or *glulb* knockdown zebrafish. The analysis revealed that there were 4042 upregulated and 3723 downregulated differentially expressed genes (DEGs) among the transcripts in *glula* knockdown zebrafish, while *glulb* knockdown zebrafish exhibited 1055 upregulated and 1759 downregulated DEGs (Figure 6A, Supplementary Table S2). The functions of DEGs were elucidated through GO analysis and KEGG analysis, aiming to identify the most significant biochemical, metabolic, and signal transduction pathways involved. The annotation results revealed a substantial number of pathways associated with metabolism. Examples encompass tryptophan metabolism, steroid hormone biosynthesis, primary bile acid biosynthesis, the PPAR signaling pathway, phototransduction, interconversion of pentose and glucuronic ester, metabolic pathways, glycolysis/gluconeogenesis, fat digestion and absorption, carbon metabolism, etc. (Supplementary Figure S3).

Numerous genes in the GLUL pathway exhibited differential expression. The knockdown of *glula* in zebrafish resulted in the downregulation of DEGs such as *glula*, *glsl*, *ppat*, *gad1b*, and *aldh5a1* and the upregulation of DEGs including *glsa*, *cad*, *gfpt1*, and *gfpt2*. Similarly, the knockdown of *glulb* in zebrafish led to the downregulation of DEGs such as *glulb*, *glulc*, *gls2a*, and *glsb* and the upregulation of DEGs *cps1* and *gfpt1* (Figure 6B). The expression of these genes in the samples is illustrated (Figure 6E). The knockdown of *glula* and *glulb* leads to the apoptosis of hair cells in zebrafish, indicating their crucial involvement in zebrafish hair cell development. By cross-referencing our previously identified gene set of hair cell marker genes [12], we discovered 106 overlapping genes among the hair cell marker gene set, the gene set resulting from *glula* knockdown in zebrafish, and the gene set resulting from *glulb* knockdown in zebrafish (Figure 6C).

The expression of these genes, influenced by *glula/glulb*, is potentially associated with hair cell development. To construct the gene interaction network, we utilized protein–protein interactions and gene expression correlation (Figure 6D and Supplementary Table S2). Among them, 15 genes (*s100s*, *anxa5a*, *smpx*, *dnajc5b*, *chrna9*, *kdm6ba*, *atp1b2b*, *atp1a1a.4*, *hebp2*, *twf2b*, *fscn2b, clic5b*, *pkma*, *ctbp2a*) were found to exhibit specific spatiotemporal expression in neuromasts or otic vesicles based on the database (http://zfin.org/) (accessed on 1 April 2024) (Figure 6F). These genes are modulated by *glula* and *glulb* and closely correlate with hair cell development. They represent potential target genes for future investigations.

## 3. Discussion

Glutamine synthetase, a pivotal enzyme in nitrogen metabolism, facilitates the condensation of glutamate and ammonia to synthesize glutamine [13]. Predominantly localized in the brain, kidney, and liver [14], this enzyme plays essential roles in the metabolic regulation of glutamate, detoxification of brain ammonia, ammonia absorption, neurotransmitter recycling, and termination of neurotransmitter signals within the brain [15]. While the existing literature has extensively explored the functions of the *Glul* gene in various aspects, such as brain and liver ammonia metabolism, cell proliferation, cell migration, and cancer occurrence [6,7,8], its role and molecular mechanism in hair cell development and regeneration remain unexplored.

In this study, in situ hybridization (ISH) analysis of whole zebrafish embryos revealed specific expression patterns of *glula* and *glulb* within the inner ear and hair cells, indicating their potential involvement in the regulation of growth and development processes associated with these structures in zebrafish. However, *glula* was not detected in the hair cells and inner ear. We investigated the functional role of *glula/glulb* via expression knockdown. The knockdown of *glula* expression resulted in a decreased survival rate and an increased teratogenic rate in zebrafish. Furthermore, the auditory organs of zebrafish were adversely affected, exhibiting abnormal otolith development and impaired hair cell function. The experimental findings from *glulb* knockdown experiments revealed a diminished response to acoustic waves in zebrafish, concomitant with the deletion of hair cells and the presence of apoptosis signals. However, supplementation of glutamine failed to rescue the reduced zebrafish hair cell phenotype following *glulb* knockdown, indicating that hair cell apoptosis resulted from the accumulation of upstream substrates. By analyzing the transcriptome data of zebrafish after *glula* and *glulb* knockdown, we found that *glula* and *glulb* knockdown can cause significant changes in marker genes involved in hair cell development and function, suggesting that *glula* and *glulb* directly or indirectly regulate hair cell development in zebrafish. In summary, this study is the first to confirm that *glula* and *glulb* regulate hair cell formation and hearing function during zebrafish development, suggesting that *glula* and *glulb* are potential deafness genes.

Transcriptome sequencing was performed on zebrafish with *glula* and *glulb* knockout, followed by analysis of the distribution and screening of differentially expressed genes (DEGs) induced by *glula* and *glulb* knockout. The KEGG analysis of the DEGs revealed enrichment in multiple metabolism-related signaling pathways, suggesting that the decreased expression of *glula/glulb* impacted various metabolic pathways in zebrafish. Meanwhile, an intersection between DEGs and hair cell-specific marker genes was identified. These genes demonstrate intricate patterns of expression correlation and protein interaction relationships. Further analysis revealed that 14 of these genes exhibited expression in the inner ear or neuromast. Among them, the SMXP gene is responsible for encoding a small protein comprising 88 amino acids, and dysfunction of this protein can lead to X-linked deafness 4 (DFNX4), a sex-linked non-syndromic hearing loss [16]. Most of these genes are involved in the development and functioning of hair cells, including *s100s*, *chrna9*, *fscn2b*, and *clic5b*. S100 proteins are calcium-binding proteins that regulate various processes [17] and participate in sensory hair cell synapse with other genes [18]. The chrna9 gene encodes a component of the acetylcholine receptor found in hair cells of the inner ear [19]. Fscn2b is specifically localized to stereocilia [20]. Deficiency of clic5b leads to impaired ciliogenesis in embryos, resulting in phenotypes associated with ciliopathies such as ventral body curvature, otolith deposition defects, altered left–right asymmetry, hydrocephalus formation, and pronephric cysts [21]. The *anxa5a* gene is one of the top differentially expressed genes in adult zebrafish inner ear hair cells [22], and *dnajc5b* is one of the top differentially expressed genes in outer hair cells [23]. The *atp1b2b* gene could affect hearing by regulating ATP production and purine metabolism in a synergetic way with cdh23 [24], suggesting their potential direct or indirect interactions with *glula/glulb*, thereby contributing to the regulation of zebrafish hair cell development.

This study provides new evidence for the linkage between glutamine synthetase and hair cell development. Still, the exact mechanism under which glutamine synthetase affects hair cell development is unclear. Meanwhile, our results indicate that glutamine could not rescue hair cell apoptosis caused by *glulb* knockdown, suggesting that hair cell apoptosis might not be caused by the decrease in glutamine levels. It might be due to the excessive ammonia in the hair cell leading to apoptosis, and it is necessary to continue to explore the levels of glutamate and glutamine in the hair cell after knocking out *glula* and *glulb*. In conclusion, our study highlights the necessity of *glula* and *glulb* for hair cell development and function, providing new targets for the clinical treatment of patients with deafness and balance defects caused by GLUL alterations.

## 4. Materials and Methods

### 4.1. Zebrafish Embryos

This study utilized three distinct zebrafish (*Danio rerio*) lines: the wild-type AB lines, the transgenic lines *Tg(SqeET10:mGFP)*, and the transgenic lines *Tg(Brn3c:mGFP)*. In the *Tg(SqeET10:mGFP)* lines, neuromasts exhibited specific expression of a membrane-bound green fluorescent protein (GFP), while in the *Tg(Brn3c:mGFP)* lines, hair cells displayed selective expression of a membrane-bound GFP. Zebrafish were bred and maintained at a constant temperature of 28.5 °C following established protocols outlined in our previous publication [25]. All animal experimentation conducted in this research was approved by Nantong University’s Animal Care and Use Committee.

### 4.2. Whole-Mount In Situ Hybridization Technique

Whole-mount in situ hybridization (WISH) was performed according to standard protocols, with PCR-amplified DNA fragments of *glula*, *glulb*, *glulc*, and *eya1* genes inserted into the pGEM-T-easy vector using specified primers (Supplementary Table S1). The Digoxigenin-labeled antisense probes of the three genes were generated through in vitro transcription using linearized pGEM-T-easy vectors containing *glula*, *glulb*, *glulc,* and *eya1* fragments along with the DIG RNA Labeling Kit (SP6/T7) from Roche (#11175025910, Indianapolis, IL, USA). Subsequently, zebrafish embryos at various developmental stages were incubated overnight with the respective probes, followed by the detection of the hybridization signal using an alkaline phosphatase (AP)-conjugated anti-digoxigenin antibody (Roche, #11093274910). Zebrafish tissues exhibiting a specific expression pattern of *glula*, *glulb*, *glulc*, and *eya1* genes were subjected to staining by the addition of an alkaline phosphatase (AP) substrate solution containing NBT/BCIP (Roche, #11681451001, Indianapolis, IL, USA) to the reaction system.

### 4.3. Morpholino Microinjection and Its Efficiency Validation

Specific splicing–blocking morpholinos targeting *glula* and *glulb* were custom-synthesized using Gene Tools (Supplementary Table S1). The morpholinos were prepared at a concentration of 0.3 mM and subsequently injected into one-cell-stage embryos. The effectiveness of *glula* morpholino in inducing mis-splicing at the junctional site of exon 2 and intron 2, as well as the efficacy of *glulb* morpholino in causing mis-splicing at the junctional site of intron 1 and exon 2, were confirmed using PCR. RNA was extracted from embryos and transcribed into cDNA. Designed primers for *glula* and *glulb* (Supplementary Table S1) were employed to assess the knockdown efficiency.

### 4.4. Synthesis of sgRNA/Cas9 mRNA, Microinjection, and Identification of Mutants

To generate zebrafish mutants with a *glulb* gene knockout, we designed a specific single-guide RNA (sgRNA) that targets exon 1 of *glulb*, following the methodology outlined in our previous publications [26,27]. The sgDNA was amplified using forward primers encompassing the *glulb* gene-targeting region and a universal reverse primer (Supplementary Table S1), with the pT7 plasmid serving as the PCR template. Subsequently, sgRNA was transcribed in vitro from sgDNA using the MAXIscriptTM T7 Transcription Kit (Invitrogen, #AM1314, Waltham, MA, USA) following the manufacturer’s instructions. Microinjections of approximately 2 nL were performed on single-cell-stage zebrafish embryos, delivering a mixture of Cas9 mRNA at concentrations of 300 ng/mL and sgRNA at 100 ng/mL. To ascertain the occurrence of a mutation, randomly selected embryos post-injection were utilized to extract genomic DNA, followed by amplification using specifically designed primers (Supplementary Table S1). Subsequently, the resulting fragments encompassing the target site were subjected to a sequencing analysis to discern the various types of mutations.

### 4.5. mRNA Synthesis and Rescue Experiments

The *glulb* DNA fragments were synthesized via PCR using the primers (Supplementary Table S1). The amplified fragments were subsequently integrated into an artificial pCS2þ vector to generate the recombinant plasmid of *glulb-pCS2þ*. The *glulb* mRNA was transcribed in vitro using the linearized recombinant plasmid as a template, employing the mMESSAGE Mmachine^TM^ SP6 Kit (Invitrogen, #AM1340), and purification of the synthesized mRNA was conducted using the RNeasy Mini Kit (Qiagen, #74104, Hilden, Germany). The solution containing 50 ng/mL *glulb* mRNA was co-injected into one-cell-stage embryos, along with sgRNA or morpholino for rescue experiments, at a volume of approximately 2 nL.

### 4.6. Startle Response

The acoustic stimulation protocol was adapted from previous studies [25]. The larvae at 5 dpf were transferred from petri dishes to wells containing 1 mL of E3 medium. Both control and *glulb*-MO zebrafish were included in the experimental group. The experimental protocol involved a 10-minute acclimation period, followed by nine acoustic or vibrational stimuli (DanioVision intensity setting) with a 20-second interstimulus interval (ISI). The variable of interest for assessing the startle response was the maximum velocity (mm/s) measured at 1-second intervals, as this parameter is most suitable for capturing the transient burst of activity associated with the startle response. Subjects with a response below 15 mm/s to the initial stimulus were excluded from the analysis. 

The vibration stimulation startle reflex was performed in accordance with the previously described protocol [28]. The larvae at 5 dpf were placed in a thin layer of culture media in a petri dish affixed to a miniature vibrator. The response of larvae to sound stimulus generated by the vibrator was captured using an infrared camera positioned above, and the recording spanned a duration of 6 s. The mean displacement and maximum velocity were employed to quantify the startle response.

### 4.7. Immunofluorescence Staining

The TdT-mediated dUTP nick end labeling (TUNEL) assay was meticulously performed according to the manufacturer’s specifications (Alexa Fluor 640, cat#: 40308ES20, YEASEN Biotech Co., Ltd., Shanghai, China) to accurately identify and quantify cell death within the hair cells of neuromasts. The zebrafish embryos were anesthetized and subsequently fixed using 4% paraformaldehyde. Following three thorough washes with PBST, the embryos were treated with 20 μg/mL proteinase K (Roche). Following this, the embryos were exposed to Alexa Fluor 640-12-dUTP Labeling Mix for a minimum duration of 3 h to achieve efficient labeling of apoptotic cells. The nuclear labeling was performed using DAPI staining. High-resolution images were acquired using a Nikon confocal microscope A1R at 40× magnification and subjected to analysis via Nikon A1R NIS Elements software (version 5.20.00). Exposure settings were meticulously optimized to minimize oversaturation.

### 4.8. Imaging and Statistical Analysis

The stereomicroscope (Olympus, MVX10, Tokyo, Japan) was employed for recording WISH experiment results. To visualize the HC phenotype in *glula* knockdown, *glulb* knockdown, and knockout models, as well as for staining and immunofluorescence analysis, a confocal microscope (Nikon A1-DUT, Tokyo, Japan) was utilized. The larvae were initially anesthetized using tricaine MS-222 (Sigma, #A5040, St. Louis, MO, USA) and subsequently embedded in 0.6% low-melting-point agarose for imaging purposes. Acquired confocal images were processed via Imaris X64 software (version 9.0.1). The experiments were meticulously repeated a minimum of three times, and the data were presented as the mean ± SEM. Statistical analysis was performed using GraphPad Prism 8.3.0 software, employing one-way ANOVA, unpaired Student’s t-tests, and two-way ANOVA to determine statistical significance. The statistical significance was determined by a *p*-value < 0.05, indicated by symbols *, **, ***, and **** above the bars representing *p*-value < 0.05, *p*-value *<* 0.01, *p*-value *<* 0.001, and *p*-value < 0.0001, respectively. Conversely, “ns” denoted no significant difference.

### 4.9. Glutamine Treatment

Diluted glutamine (50 ng/μL) was supplemented into the E3 culture medium for the cultivation of zebrafish embryos, while *glulb*-MO was injected into zebrafish embryos at the 1–2 cell stage to evaluate their developmental progression. The injections were performed at a temperature of 28.5 °C, and the developmental stages were recorded using a bright-field microscope at around 72 hpf.

### 4.10. Transcriptome Sequencing and Analysis

Transcriptome sequencing was performed to elucidate the changes in gene expression resulting from the knockdown of *glula* and *glulb* in zebrafish. Three-day-old wild-type zebrafish and zebrafish injected with *glula*-MO and *glulb*-MO were prepared for analysis, with three independent replicates performed for each treatment. The total RNA extraction was performed using TRIzol reagent (Invitrogen, Waltham, MA, USA) according to the manufacturer’s protocol. Subsequently, the quality and quantity of total RNAs were assessed using a Bioanalyzer 2100 and RNA 6000 Nano LabChip Kit (Agilent, Santa Clara, CA, USA), ensuring that the RIN values exceeded 7.0. The RNA samples were subsequently submitted to GENEWIZ Science (Suzhou, China) for deep sequencing using an Illumina Hiseq2500 platform (San Diego, CA, USA).

The DESeq2 algorithm was employed for transcript-level differential expression analysis of RNA-seq data [29]. Differentially expressed genes (DEGs) were identified based on a log2 fold change >1 and a *p*-value < 0.05. To annotate the Kyoto Encyclopedia of Genes and Genomes pathways (www.kegg.jp/kegg/kegg1.html, accessed on 1 January 2024) and Gene Ontology (www.geneontology.org, accessed on 1 January 2024), we utilized the R package Clusterprofiler [30].

The gene–gene correlation network was constructed based on the correlations of gene expression and protein interactions. Gene expression correlations were calculated using the Pearson correlation coefficient (|*r*| > 0.8, *p* < 0.05), while protein interactions were obtained from the String database [31]. The network visualization was performed using Cytoscape software (Version 3.9.1) [32].

### 4.11. Ethical Statement

All zebrafish experimentation was carried out following the NIH guidelines for the care and use of laboratory animals (http://oacu.od.nih.gov/regs/index.htm, accessed on 1 January 2024) and ethically approved by the Administration Committee of Experimental Animals, Nantong University, Jiangsu Province, China (Approval ID: 20190606-001).

## Data Availability

The original contributions presented in this study are included in the article/Supplementary Materials. Further inquiries can be directed to the corresponding authors.

## Supplementary Materials

The following supporting information can be downloaded at: https://www.mdpi.com/article/10.3390/ijms252111561/s1.
