# Supplementary material for: The Glutamine Synthetases Are Required for Sensory Hair Cell Formation and Auditory Function in Zebrafish
Source: Int J Mol Sci. 2024 Oct 28;25(21):11561. doi: 10.3390/ijms252111561 (PMC11546858; doi:10.3390/ijms252111561)
Supplement: Supplementary file 1 [file ijms-25-11561-s001.zip › Supplemental Figures-Dong.pdf]

Supplementary Figures

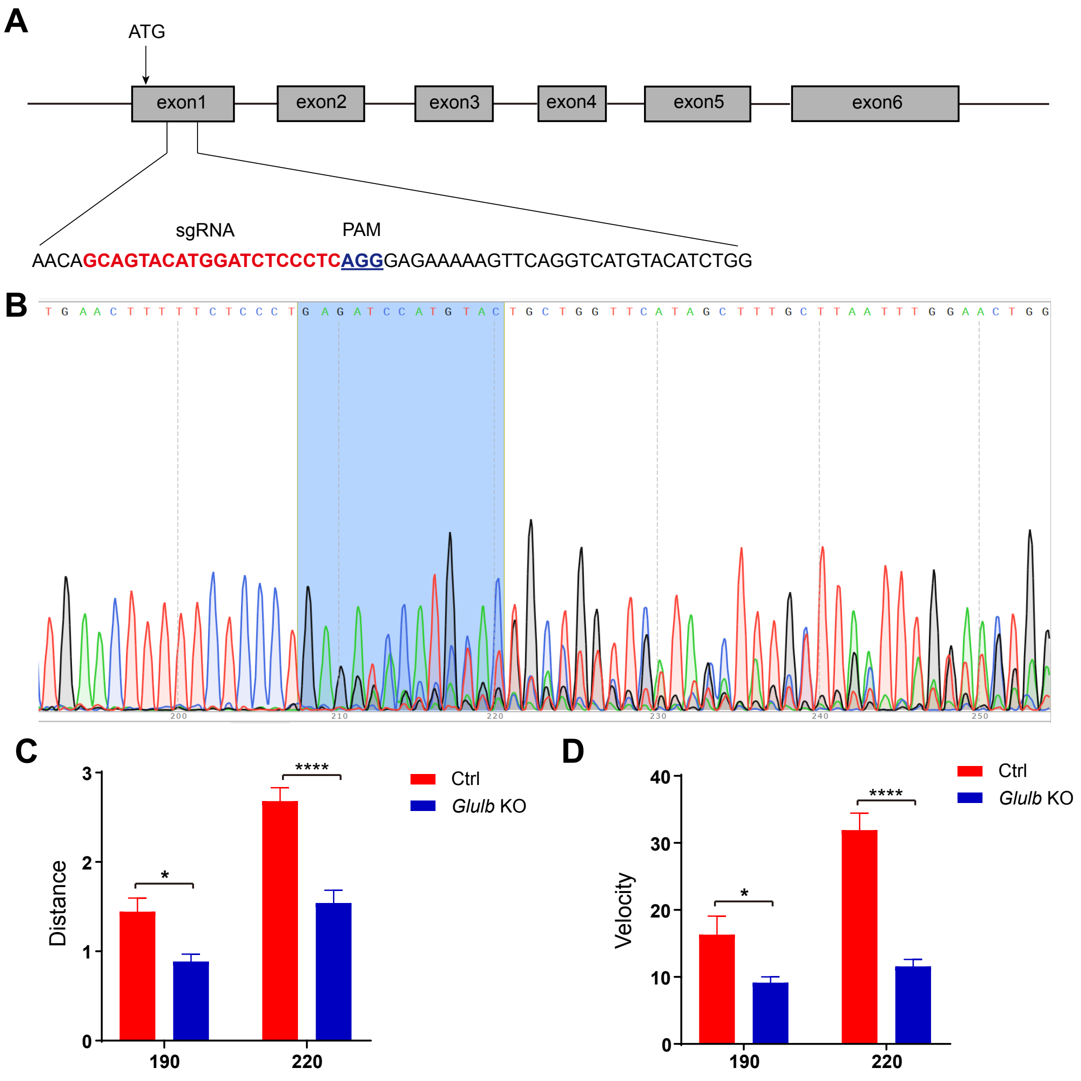

Supplementary Figure S1. The knockout of *glulb* resulted in a delayed response of zebrafish to both vibrations.

(A) The sgRNA sites were designed to specifically target the first exon of *glulb*. (B) Sequencing results of zebrafish embryos following gene knockout were obtained. The behavioral analysis of zebrafish larvae at 7 days post-fertilization (dpf) in response to vibration stimuli with intensities of 190 and 220 revealed that (C) the locomotor activity of *glulb* knockout zebrafish exhibited a significant decrease, and (D) their swimming speed was significantly reduced.

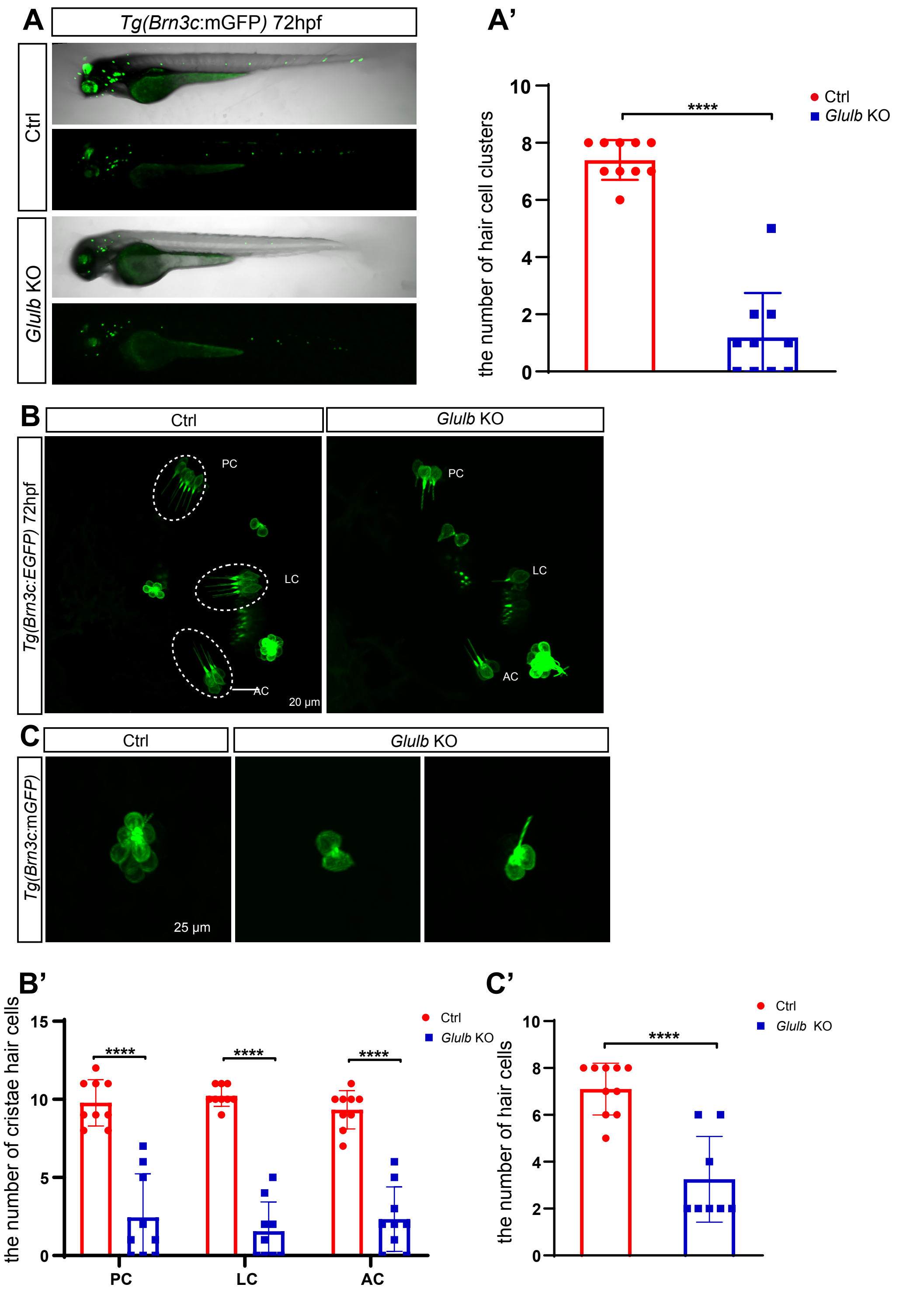

Supplementary Figure S2. The number of hair cells is diminished in *glulb* knockout zebrafish embryos.

The number of hair cell clusters in (A) the lateral line, (B) inner ear hair cells, and (C) L3 neuromast hair cells was significantly reduced in *glulb* KO zebrafish embryos compared to the Ctrl group. (A', B', C') Statistical analysis revealed significant differences.

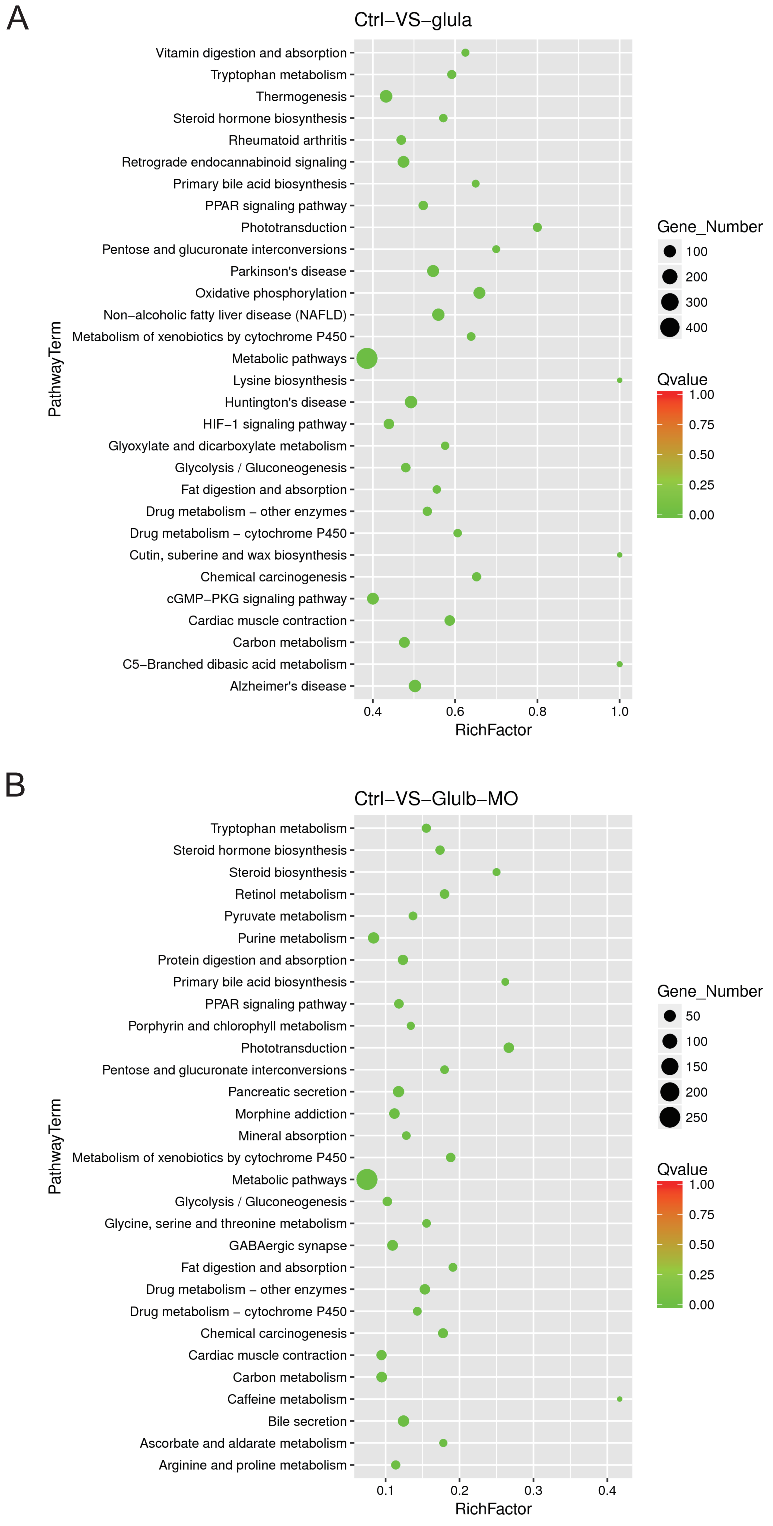

Supplementary Figure S3. KEGG enrichment annotations were performed on the transcriptome of *glula* knockdown zebrafish and *glulb* knockdown zebrafish.

Supplementary Table S1. The primer sequences of the genes investigated in this study are provided.

Supplementary Table S 2. Differentially expressed genes in the zebrafish transcriptome following knockdown of *glula* and *glulb*, as well as the gene-gene pair within the network.
